# 558 Implementation of a Nurse Driven Approach to Early Mobilization of a Cultured Epidermal Autograft Patient

**DOI:** 10.1093/jbcr/irae036.192

**Published:** 2024-04-17

**Authors:** Emily Snyder, Kara M Kastner

**Affiliations:** Medical City Plano, Plano, TX; Medical City Plano, Dallas, TX; Medical City Plano, Plano, TX; Medical City Plano, Dallas, TX

## Abstract

**Introduction:**

Early mobilization in those who suffer burn injuries is a vital part of recovery. Early mobilization can decrease length of stay, promote independence, minimize weakness and increase range of motion (ROM) (McGillicuddy et al., 2022). The use of cultured epidermal autograft (CEA) can often limit early mobilization in the burn patient due to the risk of graft loss/shearing. A nurse driven early mobilization protocol was implemented on a single patient with 45% TBSA who received two rounds of CEA.

**Methods:**

Nurse driven early mobilization was completed on a single patient after the risks/benefits were discussed with the burn physician. Preceding the surgical application of the CEA, a collaborative multidisciplinary approach was taken to create a mobilization schedule for the patient.

The Burn Educator (BE) provided 1:1 early mobilization education, a daily patient schedule, and a helpful tip handout for the RN team to reference with a goal of preventing graft loss/shearing.

The first stage of CEA was placed to the patient’s back and thighs. Beginning on post-operative day (POD) four, the Burn Nurse Supervisor, BE, and bedside RN began mobilizing per the schedule. The patient was assisted to the edge of the bed (EOB) utilizing an inflatable positioning mat. The patient would then stand and walk inside the room. During the drying time, the patient would alternate positions between sitting EOB, chair, and/or prone in the bed with the assistance of the RN or therapy team. On POD six, the patient returned to the operating room for additional CEA placement to bilateral arms. The patient continued to mobilize every day after POD six, increasing his standing and walking time each day.

**Results:**

Early Mobilization of this patient did not compromise the integrity of the CEA. Some slight sheering to the back on one thigh was noted due to the patient moving up in the chair without RN assistance. The patient was discharged home from the hospital after 48 days, not requiring transfer to a rehabilitation facility.

**Conclusions:**

In this single case study review, it was concluded that with proper education and collaboration with a multidisciplinary burn team, a nurse driven early mobilization protocol resulted in no significant graft loss/sheering. Additionally, it aided in the prevention of complications associated with prolonged immobility, allowing the patient to discharge home and eliminating the need for inpatient rehabilitation.

**Applicability of Research to Practice:**

Early mobilization can be applied to patients who have received CEA without risking harm to the graft sites. However, clear communication and planning is necessary to be successful.

References:

McGillicuddy, J., Bello, G., Bergeron, K., & Lee, J. (2022). 71 burn intensive care unit early mobility competency based orientation. Journal of Burn Care & Research, 43(Supplement_1), S48–S48. https://doi.org/10.1093/jbcr/irac012.074

